# Re-Valorizing Oyster-Shell Waste in Natural Hydraulic Lime-Based Mortars for Brick Substrate Applications: Performance and Durability

**DOI:** 10.3390/ma19010027

**Published:** 2025-12-20

**Authors:** Poliana Bellei, Manuel Francisco Costa Pereira, Isabel Torres, Genevieve Foray, Inês Flores-Colen

**Affiliations:** 1 CERIS, Instituto Superior Técnico, University of Lisbon, 1014-001 Lisbon, Portugal; ines.flores.colen@tecnico.ulisboa.pt; 2 CERENA, Instituto Superior Técnico, University of Lisbon, 1014-001 Lisbon, Portugal; mfcp@tecnico.ulisboa.pt; 3 CERIS, University of Coimbra, 3030-790 Coimbra, Portugal; itorres@dec.uc.pt; 4 Itecons, 3030-289 Coimbra, Portugal; 5 MATEIS, INSA-Lyon, UCB Lyon 1, CNRS, MATEIS UMR5510, 69621 Lyon, France; genevieve.foray@insa-lyon.fr

**Keywords:** waste recycling, sustainable mortar, aquaculture, mollusks, circular economy, blue economy

## Abstract

The re-valorisation of oyster-shell waste offers a sustainable pathway for producing eco-efficient construction materials. This study investigates the physical, mechanical, and durability performance of natural hydraulic lime (NHL) mortars incorporating oyster shells (OSs), applied to solid bricks representative of historical masonry. Two formulations were developed: one with 24% replacement of NHL by oyster-shell powder (OSP, <150 µm) and another with 30% substitution of sand by oyster-shell aggregate (OSA, 0–4 mm), both compared with a control mortar. Mortars were tested in standard molds and directly applied to bricks, including under accelerated aging conditions (temperature and humidity cycles). Results revealed that shell-incorporated mortars applied to bricks exhibited higher bulk density and compressive strength, and lower porosity, capillary water absorption, and water vapor permeability, compared with mold-cast samples. The performance for the shell-based mortars highlights the substrate–mortar interaction, consistent with the behavior of traditional lime-based systems, and the microscope characterization (poro-Hg and X-ray tomography). Shell-incorporated mortars retained stable properties after aging, with variations below 10% compared to unaged mortars. These findings demonstrate the feasibility of oyster shells as partial replacements for lime and sand, confirming its potential as an eco-efficient strategy for sustainable mortars in conserving and rehabilitating historic masonry buildings.

## 1. Introduction

**Figure 1:**
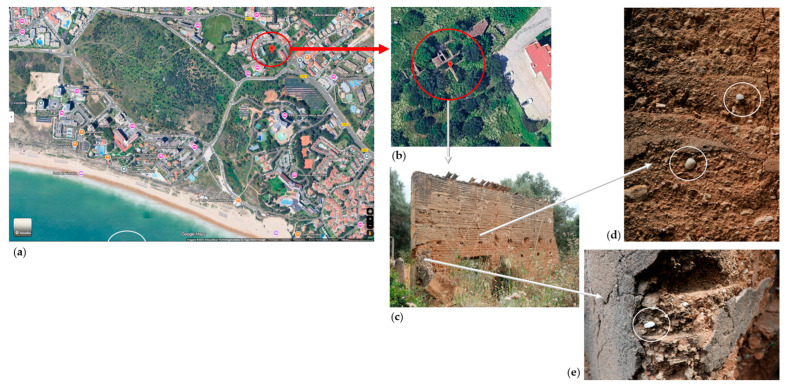
Location and wall with shells in the city of Alvor: (**a**) location of the wall near the sea; (**b**) approximate location of the wall; (**c**–**e**) shells found on the wall from the construction period.

The use of slaked lime mixed with shells and other natural aggregates has a long tradition across Europe and Asia, reflecting early sustainable construction practices [1]. For instance, in the southern region of Portugal, photographic records from the 1950s show that the roofs of Fuseta’s historic center included insulating layers composed of medium-grain sand and compacted cockle shells [2]. Shell fragments are also visible in ancient wall structures still standing in Alvor, reflecting a strong coastal building tradition (Figure 1). In Évora’s historic center, the Common Barn, built in early 1773, features a lanceolate arch portal adorned with shell ornaments as decorative elements [3]. More recently, microscopic analysis of mortars from Alcácer do Sal confirmed the presence of shell inclusions within a homogeneous matrix of lime nodules, ceramic aggregates, and a large shell fragment [4]. Furthermore, the use of seashells as a construction material was a common practice in coastal regions of China due to the abundance of limestone from shells and the need for waste management [5,6]. In Macau, the mix of available materials, such as clay, sand, rice straw, stones, and oyster shells (locally known as chunam or chunambo), was used to build the city’s first wall in 1569 [1]. These examples highlight the historical integration of marine-derived materials in lime-based mortars, offering valuable insights for modern restoration and sustainable material design.

Over the centuries, these structures have acquired heritage value, both for their antiquity and for embodying vernacular construction techniques adapted to locally available resources (shell mortar). Consequently, they are recognized as part of China’s cultural heritage [6,7]. However, due to natural degradation, the restoration of these ancient buildings is crucial to preserve their historical aesthetics and to ensure their stability and long-term durability. Through laboratory tests, Shao et al. [7] have identified suitable compositions for restoring ancient buildings made from traditional Chinese hydraulic lime (ginger nut, aga clay, and shell lime). Studies have developed oyster-shell-ash mortar with a composition similar to the ancient mortar used in historical buildings, aiming to strengthen masonry walls by partially replacing the original mortar (lime-clay mortar) [8]. Other researchers have studied shell lime mortar with the addition of organic substances and suggest its potential use as a substitute for common lime in the construction industry [6]. Therefore, drawing inspiration from traditional construction techniques in coastal regions, this study explored the incorporation of oyster shells into hydraulic lime-based mortars.

Recent studies investigating the use of oyster shells in cementitious composites have focused on factors such as particle size [9,10,11,12,13] and have considered a range of shell species, including cockles, oysters, and clams [14,15].

Nevertheless, when research is conducted on the development of mortar compositions, these activities are typically carried out under controlled laboratory conditions. Their various properties are evaluated according to applicable standards, which recommend producing standardized test samples, casting them in laboratory molds, and subjecting them to specific curing conditions. However, in practice, mortars are applied to substrates of different materials and under varying conditions, often differing from those encountered in a laboratory environment.

Some studies have explored the behavior of mortars under real conditions to assess the influence of porous substrates on their characteristics. Researchers have concluded that mortars hardened in molds exhibit different properties compared to those applied directly to substrates [16,17,18,19,20,21]. For example, Soares et al. [20] analyzed the application of aerial and natural hydraulic lime with fine sand and standard sand on solid ceramic bricks and limestone slabs, comparing their behavior with the same mortars cast in laboratory molds. The conclusions indicated that substrates with higher porosity and water absorption by capillarity, such as solid bricks (porosity: 18.3%, and water absorption by capillarity: 0.10 kg/(m^2^.s^0.5^)), have a greater influence on the behavior of the applied mortars, particularly their capillary water absorption. The average reduction reached 42% for natural hydraulic lime mortar. The authors also concluded that substrate characteristics affect the properties of applied mortars, with the degree of influence varying mainly by binder type and the presence of water-behavior-modifying additives. Torres et al. [21] studied a laboratory-mixed cement mortar and two pre-dosed mortars—one based on aerial lime and the other on cement. Furthermore, mortar produced with cement, sand, and water demonstrated greater susceptibility to substrate-induced suction than the pre-dosed cement mortar. On the other hand, these effects can also vary depending on the test method employed [19].

Although previous studies have examined changes in mortar properties using different application conditions and artificial aging [18,20], as well as the influence of alternative preparation methods in mortars with fibers [22], research focusing on the use of these variables for oyster-shell-waste-based mortars is still limited. Therefore, this study addresses that gap by exploring the performance of natural hydraulic lime mortars incorporating oyster shells (as a partial replacement: lime or sand) when used with different types of molds (laboratory vs. solid ceramic brick substrate) and after accelerated aging.

For walls’ rehabilitation or new construction, insights from previous analyses of mortar applied to the substrate are essential. Therefore, the investigation considers the behavior of mortar cast in standard laboratory molds and also compares these results with those obtained with mortars applied directly to solid ceramic-brick surfaces (typical substrates of old buildings). All conditions were cured under controlled laboratory temperature and air humidity until the testing date. After the curing period, some mortar prototypes applied to substrates were also subjected to accelerated aging to assess durability. The production and evaluation of the characteristics of shell mortars were based on a control mortar.

In addition to the performance and durability of building materials, a sociocultural aspect can be preserved by promoting the use of local materials from coastal regions, generating economic value and providing environmental benefits, such as reducing the carbon footprint in construction and minimizing waste [23,24].

## 2. Materials and Methods

### 2.1. Materials and Their Characterization

Natural Hydraulic Lime (NHL 3.5) is a binder suitable for producing rendering mortars. Its potential applications include the rehabilitation and conservation of historic and old architectural structures, as well as new construction. Mortars produced with this type of binder are compatible with various surfaces, including new masonry made from prefabricated blocks and brick, as well as stone masonry, for both internal and external use. According to its manufacturer, this material complies with the EN 459-1 standard [25]. The sand used as fine aggregate was composed of different purchased sands with calibrated particle sizes (APAS14, APAS30, APAS60, and FPS 120).

Oyster shells, donated by a cooperative from the Algarve region (Southern Portugal), were washed with water and oven-dried at 100 °C for 24 h. After crushing and sieving, the shell particles were separated for two different applications: as an aggregate (particle size between 0 and 4 mm), obtained using a cutting mill (Retsch, SM 200, Haan, Germany), and as a powder (particle size < 150 µm), produced by milling in a mortar grinder (Retsch, RM 200) followed by sieving through a 150 µm mesh.

**Figure 2:**
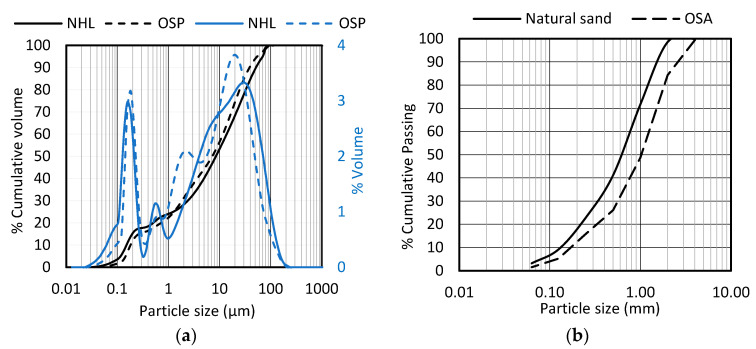
Particle size distribution: (**a**) powders: assessed by laser diffraction; (**b**) aggregates: assessed by sieve.

Figure 2a presents the particle size distribution by laser diffraction using the Malvern Mastersizer 2000 system (Mastersizer Software, V5.61) of NHL 3.5 and oyster-shell powder (OSP). Figure 2b corresponds to the particle size distribution of sand and oyster-shell aggregate (OSA) carried out according to EN 1015-1 standard [26].

**Figure 3:**
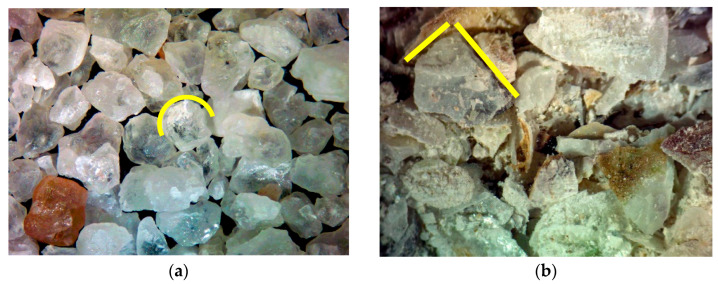
Aggregates particles representation assessed by stereomicroscopy: (**a**) natural sand, rounded grains (yellow); (**b**) oyster-shell aggregate, elongated grains (yellow).

Observations of the aggregate surfaces (sand and OSA) were made using a NIKON SMZ645 (Tokyo, Japan) stereomicroscopy. It is possible to observe that the sand particles consist predominantly of rounded grains, whereas the oyster-shell fragments display a more elongated and layered structure (Figure 3).

The bulk density of the materials was determined according to EN 1097-3 [27] using a container of known mass and volume, which was filled with material and weighed. The results were 758 kg/m^3^ and 823 kg/m^3^ for natural hydraulic lime and shell powder, respectively, and 1460 kg/m^3^ and 900 kg/m^3^ for natural sand and shell aggregate, respectively. The water absorption was also determined according to the EN 1097-6 [28] by the pycnometer method for aggregate particles passing the 4 mm test sieve and retained on the 0.063 mm test sieve. The obtained values were 0.3% for sand and 2% for shell aggregate.

Due to the binder’s compatibility with various brick types, solid ceramic-brick substrates measuring 21.6 × 10.2 × 5 cm, traditionally used in historic buildings, were selected for this study. This substrate had been previously characterized in earlier research [17,18,20] and was identified as the most influential factor in the properties of natural hydraulic lime mortar mixed with commercial sand [20].

### 2.2. Mortar Design

The control mortar was prepared with a binder-to-sand volume ratio of 1:3. In the binder replacement mix, 24% of the natural hydraulic lime was substituted with oyster-shell powder (OSP) by weight, while in the sand replacement mix, 30% of the natural sand was replaced with oyster-shell aggregate (OSA) by volume, as previously investigated [29]. The replacement levels and water content used in this study were established based on multiple substitution ratios tested [30]. That investigation evaluated different replacement levels of hydraulic lime and sand, using both identical and varied water-to-binder ratios relative to the control mortar. The selected proportions were identified as those achieving the most favorable balance between mechanical and physical performance and the maximum feasible incorporation of oyster shells, either as powder or aggregate. The water content for each mix was adjusted to achieve a target consistency of 175 ± 10 mm [31] following the specifications of CEN standards [32]. Table 1 summarizes the mix proportions and slump flow values for the control mortar and those containing OSP and OSA.

### 2.3. Mortar Condition and Test Methods

**Figure 4:**
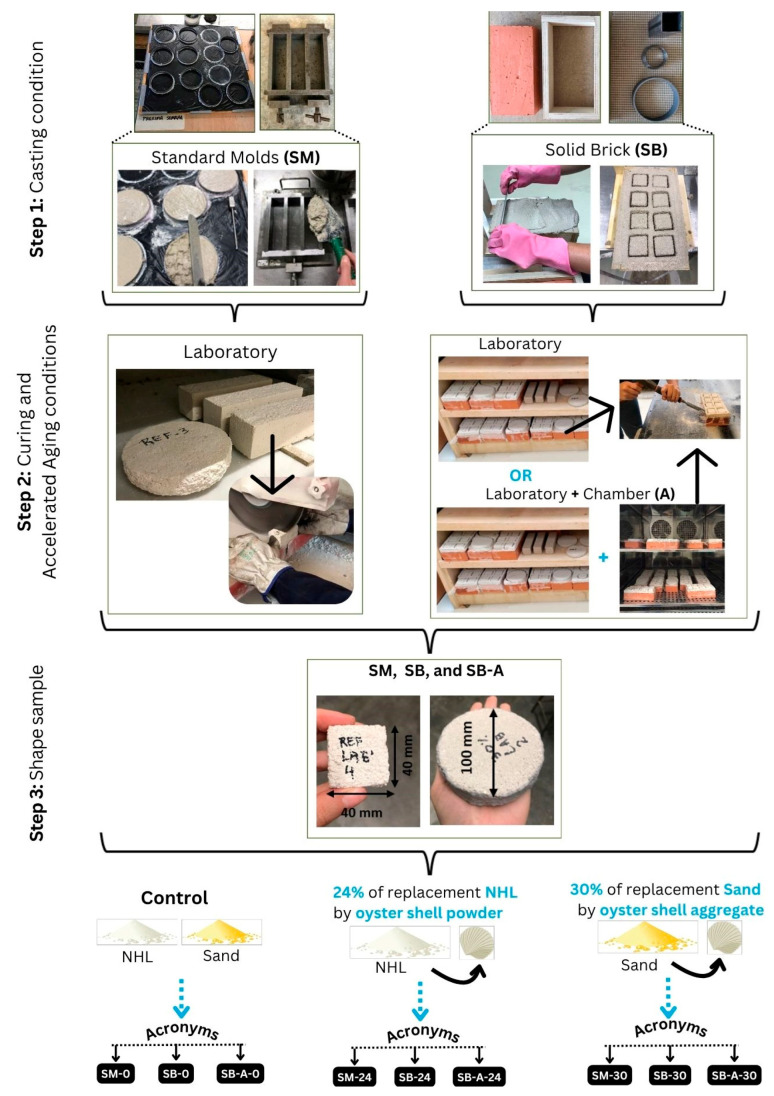
Summary of the mortars produced, casting, curing, samples, and tests.

Paragraphs (i); (ii); and (iii) explain the processes represented in Figure 4.

(i) Standard molds (SMs):

Three prismatic samples (40 × 40 × 160 mm) were cast in metal molds, and three circular samples (15 × ø100 mm) were cast in plastic molds. Then, all samples were covered with plastic wrap to keep the temperature at 20 ± 2 °C and an air humidity of 95 ± 5 % for seven days, with demolding occurring after 48 h. The samples were then stored in the laboratory at a temperature of 20 ± 2 °C and a relative humidity of 65 ± 5% until testing after 28 days. The condition of the mortar cast in standard molds and cured in the laboratory was designated: SM-0, SM-24, and SM-30 for control, 24% OSP, and 30% OSA, respectively. Immediately before the hardening state tests, the prism samples of the mortars were cut to a thickness of 15 mm using a precision saw, resulting in samples with final dimensions of 15 × 40 × 40 mm. This procedure yielded dimensions comparable to those of the mortar samples applied to and detached from the solid brick.

(ii) Solid brick (SB):

The same mortars produced to cast in SM condition were also applied to solid bricks (SBs) and cured as described in (i). For the SB condition, the process was as follows: the brick surface was slightly saturated with 100 mL of water using a spray bottle, a 5 × 5 mm fiberglass mesh was placed (this method has been previously used to facilitate the subsequent detachment of the mortar layer [33]), and a 15 mm thick layer of mortar was applied and leveled. The samples were marked with square (40 × 40 mm) and circular (ø100 mm) molds. Then, all the prototypes (mortar + brick) were covered with plastic wrap for 7 days. After 28 days, the mortar layer was carefully removed from the brick. These samples were designated as: SB-0, SB-24, and SB-30 for control, 24% OSP, and 30% OSA, respectively.

(iii) Solid brick and accelerated aging (SB-A):

In addition to laboratory curing, some SB condition prototypes were also subjected to weathering in a climate chamber (Brand: Fitoclima; Model: 1000 EC50; Supplier: Aralab (Lisbon, Portugal); Indication range: −20 °C to 150 °C/10% RH to 98%), following four cycles based on EN 1015-21 [34], with specific adaptations. After completing the aging cycles, the mortar layer was detached from the brick. SB-A-0, SB-A-24, and SB-A-30 were designated for control, 24% OSP, and 30% OSA, respectively.

The first conditioning series consisted of:

- Air temperature of 60 ± 2 °C for 8 h ± 15 min;
- Temperature of 20 ± 2 °C and air humidity of 65 ± 5% for 30 ± 2 min;
- Temperature of −15 ± 1 °C for 15 ± 15 min;
- Temperature of 20 ± 2 °C and air humidity of 65 ± 5% for 30 ± 2 min.

In the second series:

- Each sample was saturated with 300 mL of water and conditioned at an air temperature of 20 ± 1 °C and air humidity of 95% for 8 h ± 15 min;
- Temperature of 20 ± 2 °C and air humidity of 65 ± 5% for 30 ± 2 min;
- Temperature of −15 ± 1 °C for 15 min ± 15 min;
- Temperature of 20 ± 2 °C and air humidity of 65 ± 5% for 30 ± 2 min.

Tests were conducted in a hardened state, as specified in Table 2. The physical tests included bulk density, open porosity, water absorption by capillarity, and water vapor permeability. Mechanical strength was assessed through compressive strength. The tests were carried out on samples measuring 15 × 40 × 40 mm^3^, except for the water vapor permeability test, for which the sample dimensions were 15 mm × Ø100 mm. Additionally, the microstructure was analyzed for one sample from each condition, using Mercury Intrusion Porosimetry (MIP) on samples of approximately 1 cm^3^. Additionally, X-ray tomographic analysis was performed, focusing on the microstructure of the mortar with oyster shells applied to the brick with the largest particles (as aggregate).

## 3. Results and Discussion

### 3.1. Physical and Mechanical Analysis

**Figure 5:**
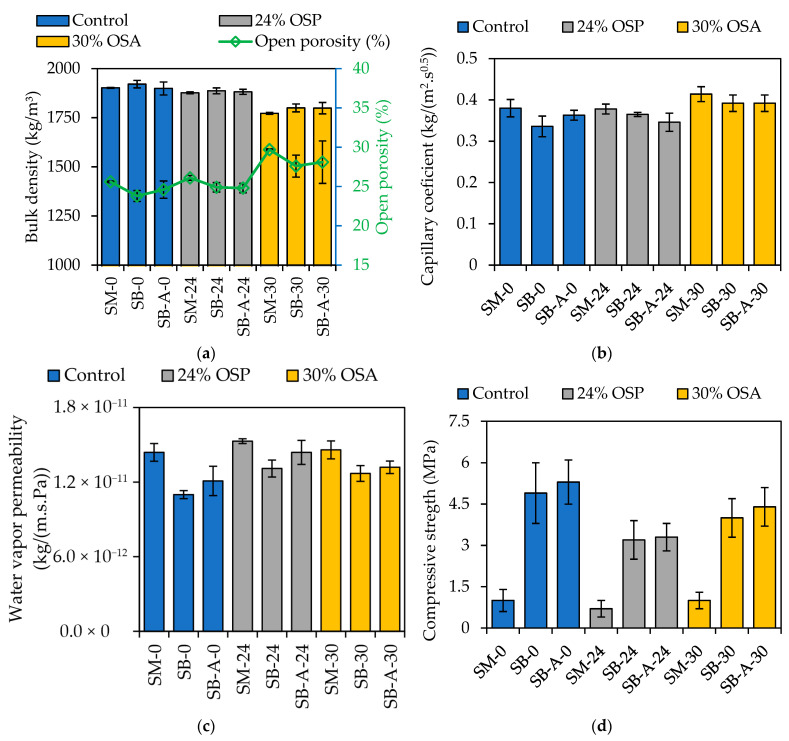
Hardened properties: (**a**) bulk density versus open porosity; (**b**) capillary water absorption coefficient; (**c**) water vapor permeability; (**d**) compressive strength.

Figure 5 summarizes the results of the physical and mechanical analysis of the mortars. For clarity, the notation used in this figure follows a consistent scheme: SM-0 refers to the control mortar cast in a standard laboratory mold, SB-0 designates the control mortar applied directly to the brick substrate, and SB-A-0 identifies the control mortar applied to the brick and subjected to accelerated aging. The same nomenclature applies to mortars incorporating 24% OSP (SM-24, SB-24, SB-A-24) and 30% OSA (SM-30, SB-30, SB-A-30).

#### 3.1.1. Bulk Density and Open Porosity

(i) Influence of oyster shell:

The results (Figure 5a) indicate that the incorporation of 24% oyster-shell powder (OSP) produces mortars with bulk density values close to those of the control mix (SM-0: 1902; SB-0: 1921 kg/m^3^), differing by only 1–2% when applied in both standard molds and solid bricks (SMs and SBs). Mortars with 30% oyster-shell aggregate (OSA) also demonstrated comparable but slightly lower bulk density values, with reductions of 7% in standard molds and 5% on brick substrates. This lower bulk density is attributed to the reduced specific weight of oyster shells (approximately 38% lower than natural sand), as reported in the literature [40]. Furthermore, their particle shape (elongated) promotes the formation of voids within the mortar matrix, resulting in a lighter composite material with greater water requirements [41].

In terms of open porosity, mortars containing 24% OSP exhibited slightly higher values than the control, with the greatest difference (approximately 5%) observed in the brick application condition (SB). However, mortars incorporating 30% OSA exhibited higher porosity, approximately 16% greater than the control, under both test conditions (SM and SB). The control mortar consistently exhibited the lowest open porosity values (SM-0: 25.6%; SB-0: 23.8%), followed by the mortar with 24% OSP and 30% OSA, which were inversely correlated with bulk density, as reported by Mesbah et al. [42]. The morphology and high absorption capacity of oyster-shell particles, as reported by Kuo et al. [43], likely contribute to the higher porosity observed, especially that which incorporates oyster shells as aggregate, compared to mortar with natural sand. Jung and Kim [13] also concluded that additional mixing water was required to achieve spreading similar to the control, attributed to the greater water-absorbing capacity of the oyster-shell aggregate, which consequently increased overall porosity.

(ii) Influence of casting and accelerated aging:

The comparison between mortars applied to the brick (SB-0: 1921; SB-24: 1887; SB-30: 1800 kg/m^3^) and those exposed to accelerated aging conditions (SB-A-0: 1899; SB-A-24: 1882; SB-A-30: 1799 kg/m^3^) revealed no significant differences in bulk density across the control, 24% OSP, and 30% OSA formulations. This stability indicates that both shell powder and shell aggregate additions did not significantly alter the mortars’ structural compactness under accelerated environmental stress, suggesting adequate resistance to density loss during aging. The control mortar applied to the brick substrate exhibited only a 1% reduction in bulk density after accelerated aging (SB-0: 1899 kg/m^3^), consistent with observations by Travincas et al. [18], who attributed such variations to material degradation during aging cycles.

Regarding open porosity, all mortars showed a slight reduction when applied to brick substrates (SB-0: 23.8; SB-24: 24.9; SB-30: 27.6%) compared to those cast in the laboratory (SM-0: 25.6; SM-24: 26.1; SM-30: 29.7%). The differences between samples applied to the brick (SB) and those exposed to accelerated aging (SB-A) conditions were lower (a maximum 3%) compared to SM and SB conditions. This suggests that substrate absorption during application exerted a greater influence on open porosity than accelerated aging. This reduction is attributed to the absorption of mixing water and fine particles by the porous substrate, which diminishes the available water in the mortar and compacts its microstructure, as supported by the literature [19,21]. Nonetheless, mortars subjected to accelerated aging exhibited higher open porosity (mainly control), reinforcing the influence of environmental cycling on this property.

#### 3.1.2. Water Absorption

(i) Influence of oyster shell:

The mortar incorporating 30% OSA exhibited the highest water absorption coefficient in standard molds (SM-30), at 0.414 kg/(m^2^·s^0.5^). Compared to the control mortar (SB-0: 0.336 kg/(m^2^·s^0.5^)), the water absorption for the 30% OSA mix (SB-30: 0.392 kg/(m^2^·s^0.5^)) represents a 17% increase in the SB condition. In contrast, the mortar containing 24% OSP displayed behavior closer to the control, showing no significant differences under standard mold (SM-24: 0.378 kg/(m^2^·s^0.5^)) conditions, and 8% increase when applied to brick (SB-24: 0.365 kg/(m^2^·s^0.5^)) compared to the control (SB-0). These results indicate that partial replacement of hydraulic lime with shell powder maintains a capillary absorption performance more comparable to the control mortar than replacement of sand with shell aggregate (Figure 5b).

The slightly lower absorption value observed for the 24% OSP mortar after accelerated aging than the control at the same condition suggests that factors beyond open porosity (SB-A-0: 0.363 and SB-A-24: 0.346 kg/(m^2^·s^0^·^5^)), such as a reduced proportion of pores in the capillary range, may influence its behavior.

Furthermore, unlike the hydrophobic mortars reported by Travincas et al. [18], which did not reach saturation after 72 h of testing, all mortars in the present study achieved water saturation during the early stages of the test. This outcome demonstrates that the non-hydrophobic nature of the natural hydraulic lime and oyster-shell mixes facilitated rapid capillary filling, resulting in stable, reproducible absorption curves.

(ii) Influence of casting and accelerated aging:

The results demonstrate a reduction in the water absorption coefficient of all mortars following application to brick substrates, regardless of composition. The control mortar showed the most pronounced decrease (12%) when comparing standard mold (SM) and brick applications (SB), while the reductions for mortars containing 24% OSP and 30% OSA were smaller, around 4–5%. These results are consistent with the observed trends in open porosity, supporting the premise that substrate suction reduces pore size and, consequently, both open porosity and water absorption [21]. Similar behavior was reported by Soares et al. [20] for natural hydraulic lime mortars containing standard and fine sands.

The mortar with 30% OSA maintained stable performance under brick applications, SB and SB-A, exhibiting no variation in water absorption (as seen for bulk density). In contrast, the control and 24% OSP mortars showed differences of approximately 7% and 5%, respectively, under the same conditions, suggesting a slightly greater sensitivity to aging effects.

The findings indicate that the influence of the brick substrate on reducing water absorption is more significant than that of accelerated aging. Moreover, incorporating oyster-shell materials, particularly at 30% sand replacement, appears to mitigate the effects of environmental aging, resulting in mortars with more stable hygric performance than the control mix.

#### 3.1.3. Water Vapor Permeability

(i) Influence of oyster shell:

The control mortar exhibited the lowest water vapor permeability among all tested formulations, followed by the mortars containing 30% OSA and 24% OSP (Figure 5c). These findings are consistent with prior studies reporting enhanced vapor and water permeability in concretes incorporating marine shells [44], attributed to their interconnected porous structure [45]. The inclusion of shell-based inert materials tends to disrupt matrix continuity, increasing the volume of capillary pores and enhancing vapor diffusion [40,46].

In the standard mold condition (SM), permeability values differed minimally between the control and 30% OSA mortars (1%), whereas the mortar with 24% OSP exhibited a 6% increase (SM-24: 1.53 × 10^−11^ kg/(m·s·Pa)).

When applied to brick substrates, the 24% OSP mortar showed a more pronounced rise (SB-24: 1.31 × 10^−11^ kg/(m·s·Pa) and SB-A-24: 1.44 × 10^−11^ kg/(m·s·Pa)), approximately 19% higher water vapor permeability than the control (SB-0: 1.10 × 10^−11^ kg/(m·s·Pa) and SB-A-0: 1.21 × 10^−11^ kg/(m·s·Pa)), probably due to a greater proportion of pores larger than 10 µm, which facilitates vapor transport [47]. The mortar containing 30% OSA demonstrated increases in permeability under applied to bricks (SB: 15%) and accelerated aging (SB-A: 9%) compared to the control. Overall, while both shell-based mortars exhibited higher vapor permeability than the control, the mix with 30% OSA showed smaller differences compared to the control, indicating that shell aggregates, despite increasing pore connectivity, may contribute to a more stable vapor transport behavior than mortar with shell powder.

All mortars presented water vapor permeability higher than that of the solid brick substrate (≈4.54 × 10^−12^ kg/(m·s·Pa) [17,18,20]), meeting the criterion established by Rodrigues [48] for compatible rendering systems.

(ii) Influence of casting and accelerated aging:

The application of hydraulic lime-based mortars, both with and without oyster-shell additions, to brick substrates (SB) reduced water vapor permeability compared with samples molded under laboratory conditions (SM). This behavior aligns with findings for pre-dosed lime and pre-dosed lime cement mortars [18,21]. The most pronounced decreases were observed in the control mortar (24%), followed by the mortars containing 24% OSP (14%) and 30% OSA (13%). The decrease in water vapor permeability observed in the mortars applied to brick substrates (SB) compared to those cast in standard molds (SMs) can be attributed to their lower open porosity. Previous studies have demonstrated a direct correlation between vapor permeability and open porosity [17,49].

The European Standard EN 998-1 [50] establishes a maximum water vapor diffusion resistance factor (μ) of 15 for mortars in renovation and thermal insulation uses. This factor, defined by the ratio between the air permeability coefficient ($\delta_{a}$ = 1.94 × 10^−10^ kg/(m·s·Pa)) and the material’s water vapor permeability (δ, Figure 5c), indicates that a lower δ leads to a higher μ. As the mortar casting in brick turns less porous, certain mortars, especially SB-0, SB-30, and SB-A-0, did not meet the standard’s required limit.

When subjected to accelerated aging (SB-A), all mortars exhibited slightly higher permeability than those cured under laboratory conditions (SB), consistent with literature values reported for aerial lime mortars [20], especially for the control mortar and the one with 24% OSP (differences of around 9%).

#### 3.1.4. Compressive Strength

(i) Influence of oyster shell:

The trends of compressive strength of the mortars are presented in Figure 5d. It should be noted that the flexural strength test was not performed due to the samples’ adapted dimensions. This test will be the subject of future research in this area.

The control mix and the mix containing 30% OSA exhibited identical compressive strength values when tested in standard molds, surpassing by 43% the performance of the mixture with 24% OSP under the same conditions. However, when applied to a substrate, the compressive strength of the control mortar exceeded that of the 30% OSA mortar and 24% OSP mortar. According to Torres et al. [21], the compressive strength of mortars is inversely proportional to their open porosity, which explains the higher strength of the less porous control mortar. However, Travincas et al. [18] did not confirm this correlation. Rato [51] emphasized that porosimetry, rather than open porosity alone, provides a more accurate explanation of the mechanical behavior of lime mortars, as it reveals pore distribution, size, and connectivity (factors that critically influence crack propagation). Therefore, despite its higher open porosity, the mortar with 30% OSA demonstrated higher compressive strength than the 24% OSP mortar. This behavior may be associated with a lower proportion of large pores, which could help mitigate stress concentration.

The reduced strength of the OSP mortar may be attributed to its lower binder content, resulting in fewer hydrated compounds and, consequently, weaker regions within the microstructure. This observation aligns with Griffith’s theory [52] which postulates that the mechanical strength of porous materials is governed by flaws acting as stress concentrators. Such imperfections facilitate crack initiation and propagation, ultimately leading to mechanical failure. Thus, the lower performance of the OSP mortar can be attributed to the interplay among binder deficiency, pore structure, and microstructural defects, which compromise its compressive strength.

(ii) Influence of casting and accelerated aging:

Mortars molded in standard molds (SMs) displayed significantly lower compressive strength compared to those applied directly to brick substrates and cured under laboratory (SB) or accelerated aging (SB-A) conditions. The control mortar and mixes containing 24% OSP and 30% OSA exhibited substantial increases in compressive strength for SB-A relative to SM: 430%, 371%, and 340%, respectively. The differences between mortars detached after 28 days of curing (SB) and those subjected to accelerated aging (SB-A) were minor, with the smallest variation (3%) observed between the SB-24 and SB-A-24 conditions. The control and 30% OSA mortars showed slightly higher differences of approximately 8% and 9%, respectively.

The compressive strength behavior of all mortars followed a consistent pattern, with significant improvement upon contact with brick substrates. This trend aligns with findings from Travincas et al. [18], who observed similar behavior in pre-dosed hydraulic lime mortars. The phenomenon is attributed to substrate absorption: as brick substrates become more porous and absorbent, suction effects reduce mortar porosity, leading to enhanced mechanical strength [21]. Among the tested compositions, the control mortar achieved the highest compressive strength, followed by the mortar containing 30% OSA and, subsequently, the 24% OSP mixture, reflecting the direct relationship between substrate interaction, pore refinement, and strength development.

### 3.2. Microstructural Analysis

#### 3.2.1. MIP

**Figure 6:**
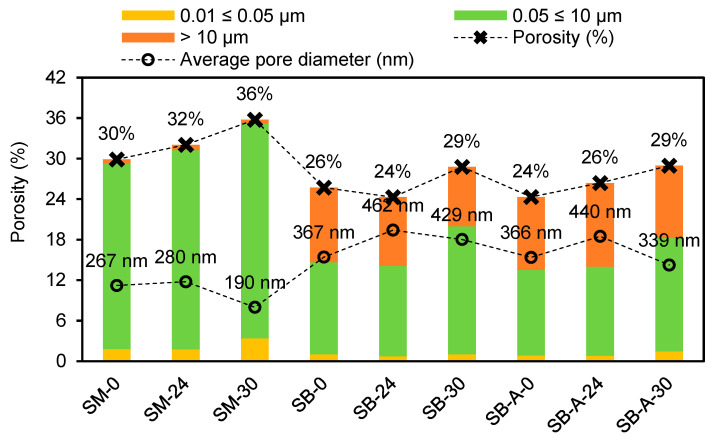
MIP measurements: porosity; average pore diameter; pore range 0.01 ≤ 0.05 µm, 0.05 ≤ 10 µm, and >10 µm.

**Figure 7:**
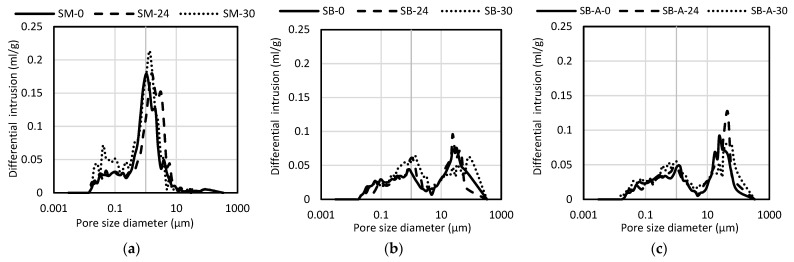
Pore diameter distribution of the control mortar and mortars containing 24% OSP and 30% OSA: (**a**) standard molds; (**b**) applied on solid ceramic brick substrates and; (**c**) applied on solid ceramic brick substrates and exposure to accelerated aging cycles.

Figure 6 presents the data from the porosimeter test. Figure 7a shows the pore size distribution of the control mortar and mortars with 24% OSP and 30% OSA cast in standard molds. Figure 7b shows the corresponding mortars applied to solid ceramic brick substrates, while Figure 7c illustrates the same mortars after exposure to accelerated aging cycles.

The intrusion porosimetry results revealed that mixes, including control mortar and mortar with 24% OSP, and conditions, applied and detached from the substrate (SB and SB-A) for all mortars, exhibited lower total porosity than those cast in standard molds (SMs), consistent with the findings on open porosity. Furthermore, the porosities measured by MIP for the mortars applied on the substrate were more similar to those measured with water penetration (Figure 5a) compared to the porosities of the SM for all mortars. The literature reported higher porosity due to the higher pressure used in the test with mercury [53,54], which may have a greater impact on the condition of SM mortars.

The influence of the substrate on pore structure is also evident. The average pore size seen in Figure 6 of the mortars under SB and SB-A conditions closely matched the average pore size of the brick substrate (≈480 nm) reported from the literature [17,18,20], contributing to the lower total pore volume measured in these samples.

Differences between mortars cast in molds and those applied to brick further highlight the role of substrate absorption. Mortars produced in standard molds exhibited smaller average pore diameters, consistent with results reported for cement mortars. The non-absorptive surface of the mold promoted complete hydration and the formation of finer binder pores, whereas the brick substrate induced suction stresses that reduced pore diameters [21]. This effect is evidenced by the higher proportion of pores in the 0.01–0.05 µm range in SM mortars compared to those on the substrate.

The impact of substrate-induced changes is also reflected in the range of pores influencing capillary transport. Pores between 0.05 and 10 µm play a critical role in water capillarity [47]. All mortars applied on brick exhibited a reduction in this pore range relative to those molded in standard forms, which corresponds to the lower water-absorption coefficients observed for mortars: SB and SB-A (Figure 5b). The correlation between reduced pore size and the suction stresses imposed by the brick substrate reinforces this relationship [21].

Finally, the proportion of larger capillary pores (>10 µm) also affects both water-vapor permeability [47] and mechanical strength [55]. As discussed in Section 3.1.3 and Section 3.1.4, the mortar with 30% OSA exhibited water vapor permeability and compressive strength values comparable to those of the control. This behavior can be associated with its lower fraction of pores exceeding 10 µm, which contributes to improved performance.

It should be noted that MIP may underestimate very fine pores due to ink-bottle effects, as mercury intrusion is governed by pore-throat size rather than cavity size.

#### 3.2.2. X-Ray Tomography

**Figure 8:**
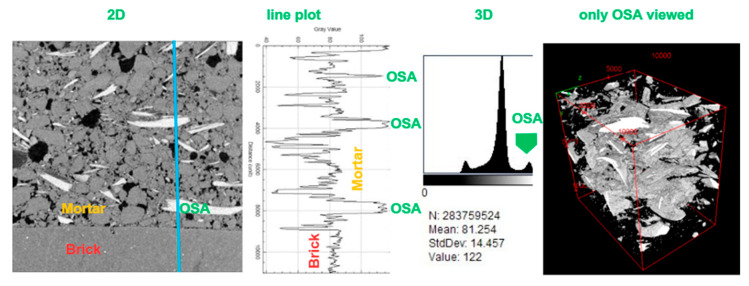
SB-30: X-ray tomography, (**left**) cross-section view and grey level profile along the vertical blue line, and; (**right**) 3D grey-level histogram and filtered 3D volume OSA viewed only (pores, binder, aggregates are translucent).

Three-dimensional X-ray tomography was used to investigate the internal microstructure of the SB-30 mortar. To better understand the spatial distribution and orientation of the shell inclusions and pores, a 3D reconstruction of the scanned volume was performed. Figure 8 shows a vertical cross-section revealing a mortar-and-brick substrate. The grey-level line plot across the sample (see blue line) confirms that with the 15 microns resolution used, each phase can be thresholded: the OSA near 105–255, the pores near 0–50. In the 2D cross-section (Figure 8, first image), high-density inclusions (oyster shells) appear white, while low-density features (air-filled pores) are dark. High values (peaks around 150) correspond to highly attenuating phases, specifically the oyster-shell fragments (CaCO_3_-rich). Low grey values (peaks around 40) represent air-filled or void regions (pores) with minimal X-ray attenuation. The intermediate grey-level phase represents the matrix, composed of a heterogeneous mix of hydration products, fine aggregates, and pores smaller than the resolution. A more refined 3D histogram across all 755 stacks confirms this. The 3D rendering (Figure 8, last image) sheds light on the preferential OSA alignment along the horizontal plane. Interstitial pores, irregular in shape and size, are uniformly distributed throughout the mortar, forming a fully interconnected void network with a volume fraction of 8.41% (see volume in the Supplementary Materials—Figures S1 and S2, and Video S1). These features have implications for the mortar’s transport properties and mechanical integrity. This corroborates the higher values of water absorption by capillarity, water vapor permeability, and lower mechanical-strength values compared to the control mortar.

**Figure 9:**
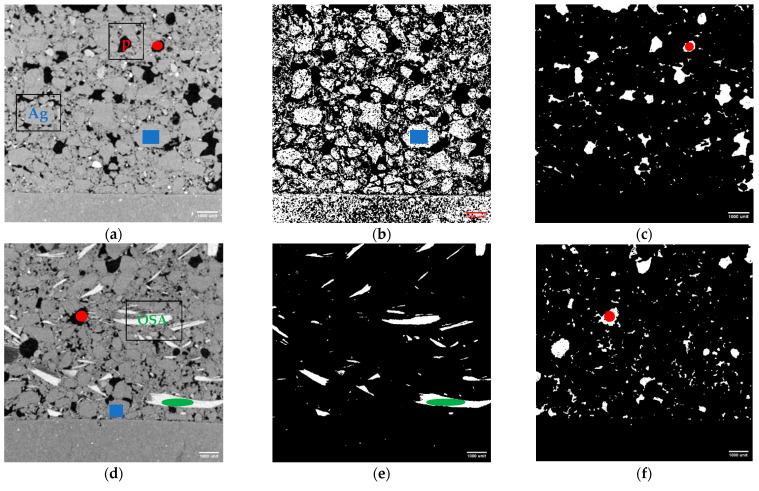
(**a**,**d**) full view; (**b**,**c**,**e**,**f**) filtered view: SB-0: (**a**) 2D cross-section, (**b**) aggregates only (blue), and (**c**) pores only (red); SB-30: (**d**) 2D cross-section, (**e**) shells aggregate (green), and (**f**) pores (red). All figures are drawn to the same scale.

Figure 9a–c shows a 2D cross-section of the control mortar, providing insight into the distribution of aggregates and pores throughout the matrix. Similarly, Figure 9d–f presents the corresponding views for the mortar containing oyster-shell aggregates, with visualization of the shell particles (Figure 9e). In both mortar types, a reduction in pore content is observed near the mortar–substrate interface (Figure 9c,f), highlighting the influence of substrate suction in this region. Furthermore, porosimetry results indicated that pores larger than 15 microns accounted for 37% of the total porosity in the control mortar (SB-0), while in the SB-30 mortar this fraction was reduced to 26%. The X-ray tomography analysis was focused on pores within this size range. The lower proportion of large pores in the SB-30 mortar may help to explain the compressive strength results found.

## 4. Conclusions

This study evaluated the performance of hydraulic lime-mortar mixes incorporating oyster-shell waste as powder and aggregate, with different water content, cast under different conditions (laboratory molds vs. brick substrates). Overall, the application of the mortars onto the brick substrate enhanced performance, increasing bulk density and compressive strength while reducing open porosity, capillary water absorption, and water vapor permeability. These improvements align with results reported in the literature for other types of mortar applied to this same solid ceramic brick substrate with high suction.

- The partial replacement of hydraulic lime with 24% OSP and of sand with 30% OSA resulted in mortars with slightly lower bulk density and mechanical strength, and higher open porosity, capillary water absorption, and water vapor permeability than the control mortar under laboratory conditions. However, most variations were within 10%, except for compressive strength in the 24% OSP mortar (30% lower) and open porosity in the 30% OSA mortar (16% higher). When applied to brick substrates, similar trends were observed, except for the additional variations (below 20%)—higher water vapor permeability for the OSP mortar and higher capillary water absorption and lower compressive strength for the OSA mortar.
- Casting mortars directly on the solid ceramic brick substrate influenced physical and mechanical properties due to the substrate’s high absorption capacity, which affected all mortar types. This effect was most pronounced in water vapor permeability and compressive strength, confirming that substrate–mortar interaction impacts performance. Microscopic analyses (Hg porosimetry and X-ray tomography) provided insights supporting these findings, highlighting how substrate absorption modifies pore structure and, consequently, mechanical and transport behavior.
- After accelerated aging, all mortars exhibited a decrease in water vapor permeability and a slight increase in compressive strength, with changes below 10%. The influence of the substrate often surpassed that of aging, emphasizing its key role in defining mortar performance, and the resistance of shell mortars to the influence of environmental cycles.

Overall, these findings demonstrate the technical feasibility of incorporating oyster-shell waste into hydraulic lime mortars. The results highlight their potential for sustainable construction applications, offering a circular approach to waste valorization and raw material reduction. Future research should further explore the environmental impact and compatibility of these mortars with other traditional masonry. This study focused on solid ceramic bricks as a representative substrate, and further investigations are needed to support the development of eco-efficient mortar systems that remain compatible with historic heritage structures.

## Figures and Tables

**Table 1:** Mix design of the control mortar and mortars with oyster shell, with their respective slump flow values.

Binder (%)	Sand (%)	OSP (%)	OSA (%)	w/b	Slump Flow (mm)
100	100	0	0	1.07	170
76	100	24	0	1.07	170
100	70	0	30	1.18	173

**Table 2:** Tests performed in the hardened state.

Test and Normalization	Notes	Number of Samples ^1^
Bulk density, EN 1636 [35]	-	SM: 5; SB: 10, SB-A: 10
Open porosity, EN 1636 [35]	-	SM: 5; SB: 10, SB-A: 10
Capillary water absorption, ISO 15148 [36]	Weighing in minutes (after the start of the test): 5, 10, 30, 60, 90, 180, 300, 480, 1440, 2880, 4320 The slope of the test curve is used to calculate the coefficient.	SM: 5; SB: 10, SB-A: 10
Water vapor permeability, EN 1015-19 [37]	Wet vat method: 20 °C (temperature); 50% (relative humidity)	SM: 3; SB: 3, SB-A: 3
Compressive strength, EN 1015-11 [38]	Instron, 2530-444 model (Norwood, MA, USA)	SM: 5; SB: 10, SB-A: 10
MIP, ISO 15901-1 [39]	High-pressure: up to 33,000 psi; Low-pressure: up to 60 psi. Micromeritics mercury porosimeter, model Autopore IV 9500 (Norcross, GA, USA)	SM: 1; SB: 1, SB-A: 1
X-ray Tomography	CT scanner v|tome|x s tomograph, manufactured by the company GE Sensing & Inspection Technologies Phoenix (Wunstorf, Germany) X-ray, its X-ray source is operated with a Tungsten cathode at a voltage of 60 kV. The detector in this setup is an ultra-fast flat-panel, Varian Paxscan (Salt Lake City, UT, USA), CCD camera with a pixel size of 15 μm.	2D: SB-0: 1; SB-30: 1 3D: SB-30: 1

^1^ Number of samples of each mortar: control, 24% OSP, and 30% OSA (exception for X-ray tomography).

## Data Availability

The original contributions presented in this study are included in the article/supplementary material. Further inquiries can be directed to the corresponding author.

## Supplementary Materials

The following supporting information can be downloaded at: https://www.mdpi.com/article/10.3390/ma19010027/s1, Figure S1: OSAmortar3D_G)OSA_R)LargeP_O)SPores; Figure S2: OSAmortar3Dcross_G)OSA_R)LargeP_O)SPores; Video S1: Volume.
