# Supplementary figures and images for: Re-Valorizing Oyster-Shell Waste in Natural Hydraulic Lime-Based Mortars for Brick Substrate Applications: Performance and Durability
Source: Materials (Basel). 2025 Dec 20;19(1):27. doi: 10.3390/ma19010027 (PMC12786797; doi:10.3390/ma19010027)

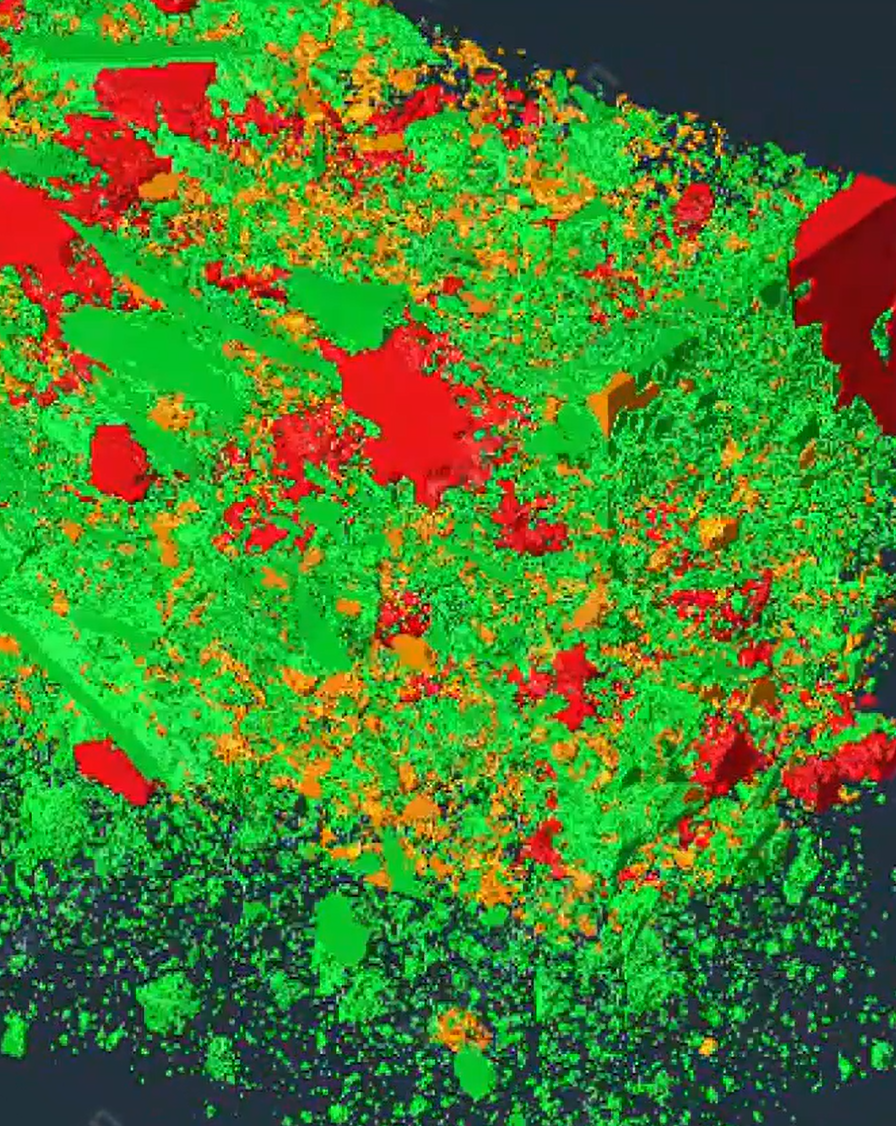

Supplement: Supplementary file 1 [file materials-19-00027-s001.zip › Figure S1 - OSAmortar3D_G)OSA_R)LargeP_O)SPores.png]

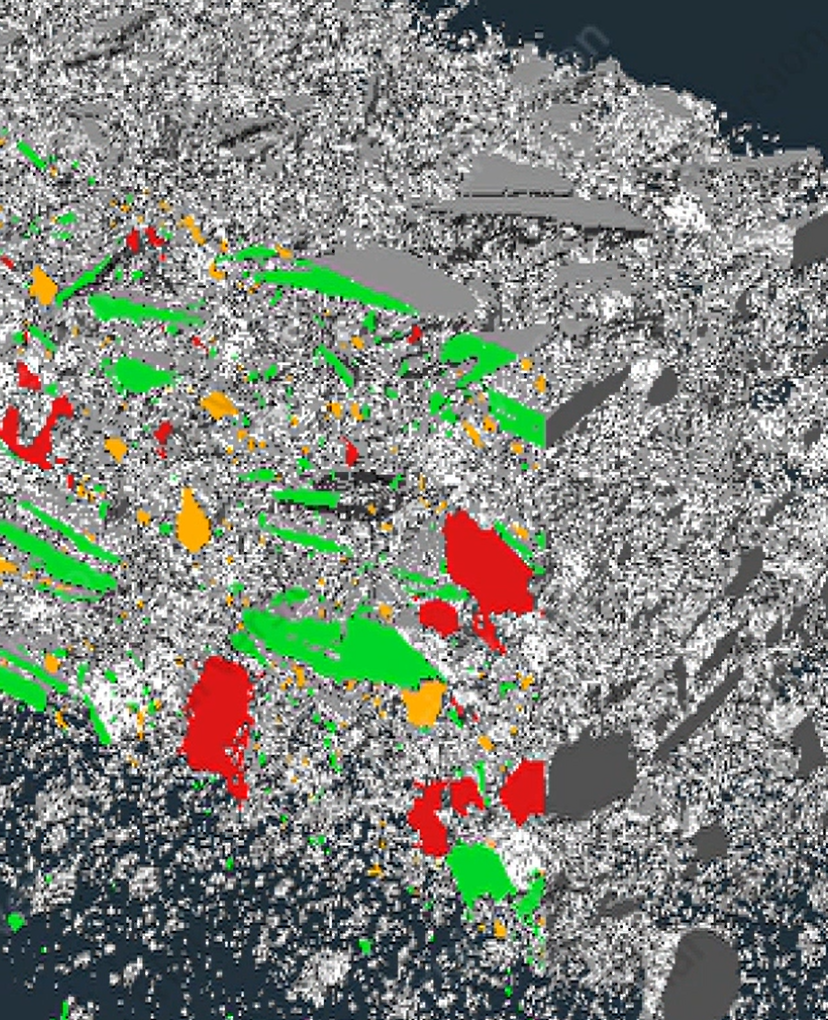

Supplement: Supplementary file 1 [file materials-19-00027-s001.zip › Figure S2 - OSAmortar3Dcross_G)OSA_R)LargeP_O)SPores.png]
